# Intranasal ketamine for acute traumatic pain in the Emergency Department: a prospective, randomized clinical trial of efficacy and safety

**DOI:** 10.1186/s12873-016-0107-0

**Published:** 2016-11-09

**Authors:** Shachar Shimonovich, Roy Gigi, Amir Shapira, Tal Sarig-Meth, Danielle Nadav, Mattan Rozenek, Debra West, Pinchas Halpern

**Affiliations:** 1 Sackler School of Medicine at Tel Aviv University, 55 Haim Levanon Street, Ramat Aviv, 69978 Israel; 2 Department of Orthopedic Surgery, Tel Aviv Sourasky Medical Center, 6 Weizmann Street, Tel Aviv, 64239 Israel; 3 Department of Emergency Medicine, Tel Aviv Sourasky Medical Center, 6 Weizmann Street, Tel Aviv, 64239 Israel

**Keywords:** Intranasal ketamine, Analgesia, Trauma, Morphine, Mass casualty

## Abstract

**Background:**

Ketamine has been well studied for its efficacy as an analgesic agent. However, intranasal (IN) administration of ketamine has only recently been studied in the emergency setting. The objective of this study was to elucidate the efficacy and adverse effects of a sub-dissociative dose of IN Ketamine compared to IV and IM morphine.

**Methods:**

A single-center, randomized, prospective, parallel clinical trial of efficacy and safety of IN ketamine compared to IV and IM morphine for analgesia in the emergency department (ED). A convenience sample of 90 patients aged 18–70 experiencing moderate-severe acute traumatic pain (≥80 mm on 100 mm Visual Analog Scale [VAS]) were randomized to receive either 1.0 mg/kg IN ketamine, 0.1 mg/kg IV MO or 0.15 mg/kg IM MO. Pain relief and adverse effects were recorded for 1 h post-administration. The primary outcome was efficacy of IN ketamine compared to IV and IM MO, measured by “time-to-onset” (defined as a ≥15 mm pain decrease on VAS), as well as time to and degree of maximal pain reduction.

**Results:**

The 3 study groups showed a highly significant, similar maximal pain reduction of 56 ± 26 mm for IN Ketamine, and 59 ± 22 and 48 ± 30 for IV MO and IM MO, respectively. IN Ketamine provided clinically-comparable results to those of IV MO with regards to time to onset (14.3 ± 11.2 v. 8.9 ± 5.6 min, respectively) as well as in time to maximal pain reduction (40.4 ± 16.3) versus (33.4 ± 18), respectively.

**Conclusions:**

IN ketamine shows efficacy and safety comparable to IV and IM MO. Given the benefits of this mode of analgesia in emergencies, it should be further studied for potential clinical applications.

**Trial registration:**

Retrospectively registered on 27 June 2016. ClinicalTrials.gov ID: NCT02817477

**Electronic supplementary material:**

The online version of this article (doi:10.1186/s12873-016-0107-0) contains supplementary material, which is available to authorized users.

## Background

Opiates are the current mainstay of severe pain relief in the pre-hospital setting as well as the Emergency Department (ED). Despite their efficacy, opiates such as morphine are inconvenient, as they necessitate either an intravenous (IV) catheter to be placed or an intramuscular (IM) injection to be administered. Intranasal (IN) administration of opiates is gaining momentum [1–3] but is still uncommon and suffers from most of the drawbacks of opiates. Opiate administration in hemodynamically-unstable patients or those with potential respiratory and airway problems, requires caution and frequent monitoring. Furthermore, administration of IM opiates can be inadequate, due to uncertainty of the extent of absorption, rendering IV opiates the current standard-of-care. The IV route is oftentimes complicated by difficult IV catheter placement in the pre-hospital, military or mass casualty setting or even during routine ED care, leading to delayed pain relief [4]. IN administration of analgesics has been studied extensively and generally found to be easy, safe and effective, especially with opiates [1–3].

Ketamine, an NMDA-antagonist has been widely studied for its efficacy in analgesia and anesthesia. However, administration of ketamine via the IN route, especially in adults, has only recently been studied and requires further elucidation in the acute setting [5, 6]. Recent studies examining the role of intranasal ketamine for analgesia in the ED, provided inconclusive results, reporting that between 56 and 88 % of patients experienced clinically-significant pain relief [5, 6]. The efficacy and side-effect profile of IN ketamine has not yet been well compared to opiates in both the ED and pre-hospital setting. Given the significant potential for a simple, safe and clinically and logistically convenient mode of analgesia for acute pain, defining IN ketamine’s safety and efficacy seems especially important. This prospective, open label, single-center, randomized clinical trial was designed to assess the efficacy and safety of IN ketamine in the ED setting.

## Methods

### Trial design

This was a single-center, randomized, prospective, open label, parallel clinical trial of efficacy and safety of IN ketamine compared to IV and IM morphine for analgesia in acute traumatic pain in the ED. A convenience sample of 90 patients aged 18–70 years experiencing moderate to severe orthopedic pain (≥80 mm on a 100 mm visual analog scale (VAS)) were randomized to receive either IN ketamine, IV morphine or IM morphine. Pain was measured on a 100 mm VAS prior to administration of analgesia and at 5 min intervals for 1 h following administration. Adverse effects were recorded. Patients were enrolled during times when a trained medical student researcher (see below) was available for data collection, on a single random day each week, during either the morning or evening shifts. These sampling times were varied to avoid any selection bias. The study took place between September 2012 and April 2014.

### Study setting and population

The study took place at a large, university-affiliated, Trauma Level I urban hospital serving a population of over 360,000 residents and over one million daily commuters. The ED serves over 140,000 adults annually. Institutional Review Board approval was received. Trained medical students collected the data, and were supported by the ED physicians, nursing staff, and orthopedic surgeons. Opiate delivery and informed consent procedures were performed and collected by licensed medical staff; IN ketamine administration and data collection was completed by the student research technicians. Research assistants performing the actual study procedure were last-year medical students who had undergone a 1 h tutorial by one of the principal investigators, involving the study aims and methodology, relevant drug pharmacology, potential side effects and complications, problem-solving and drug delivery optimization, research assistant - research subject interaction etc. During the first 3–5 procedures each research assistant was closely supervised by an experienced researcher, until deemed sufficiently proficient for independent work.

Patients aged 18–70 years, with mild to moderate blunt trauma (sustained in road, workplace and home accidents) causing moderate to severe pain (≥80 mm score on a 100 mm VAS) were eligible for participation in the study. Additional inclusion criteria were: a Glasgow Coma Score (GCS) of 15, body weight of 50–110 kg, systolic blood pressure of 90–160 mmHg, heart rate <100 bpm. Patients were also required to have an American Society of Anesthesiologists (ASA) score of 1 or 2, deny head injury, and deny regular use of opiates. Exclusion criteria included any analgesia received within the prior 3 h, allergic sensitivity to morphine or ketamine, a large meal ingested within the previous hour, pregnancy, deviated nasal septum or trauma to the nose, and a history of a psychiatric condition. Despite evidence that ketamine does not exacerbate intracranial hemorrhage in patients with head trauma [7–10], patients with head injury complaining of loss of consciousness, dizziness, vomiting, or nausea were excluded as well.

### Study materials

Ketamine Hydrochloride for Injection 50 mg/mL (Rotexmedica, Tritau, Germany) was used in the study. The atomization device used to dispense the medication was the Intranasal Mucosal Atomization Device (Wolf Tory Medical, Salt Lake City, UT, USA).

### Study protocol

All potentially eligible patients were approached upon arrival to the ED and asked for their level of pain. Patients meeting the eligibility criteria were offered participation in the study by a physician unaffiliated with the research. Once written informed consent was obtained, patients were randomized to IN ketamine, IM morphine or IV morphine, in a 1:1:1 ratio, using a randomization scheme that utilized the last digit of their Government-issued Personal Identification Numbers. Vital signs including blood pressure, heart rate, respiratory rate, and oxygen saturation were obtained, and an initial VAS reading was obtained immediately prior to administration of analgesia. Patients were then given either 1 mg/kg IN ketamine, 0.15 mg/kg IM morphine, or 0.1 mg/kg slow IV bolus of morphine. Patient weight was rounded to the nearest five kilograms, and the dose was calculated accordingly.

IN administration involved visually assuring that the nares were patent, followed by the administration of ketamine spray in 0.1–0.2 mL aliquots into each nare from a 1cc syringe at intervals of 10–30 s. Patients were instructed to report post-nasal drip of ketamine which resulted in increased intervals and augmentation of the angle and distance of the atomization device from the nare. IV morphine was administered in a slow bolus over 30–60 s. IM morphine was administered via a gluteal muscle injection by a registered ED nurse.

Vital signs and VAS measurements were obtained at 5 min intervals for 60 min. For VAS pain-assessments, patients were approached and asked to mark their pain level on subsequent 100 mm lines representing pain from 0 to 100 %. This was self-reported pain and was not dependent on the researcher. Adverse effects were recorded at the end of 1 h using the ‘Opiate Related Symptom Distress Scale’ [11] and included measurements of the presence, frequency, intensity and disruptiveness of 12 common opiate side-effects. Among these were nausea, vomiting, urinary retention, constipation, difficulty concentrating, dizziness, confusion, and others. Patients were then asked to rate overall pain relief on the VAS, and provide subjective comments if pain relief was judged by them to be inadequate.

### Outcome measures

The primary outcome was effectiveness of IN ketamine in decreasing pain intensity, compared to IV and IM morphine. This was measured by the time to achievement of a clinically meaningful pain reduction (defined as 15 mm pain reduction on the VAS), degree and time to maximal pain reduction. A value of 13 mm VAS score reduction was previously shown to be clinically-significant [12], and our choice of 15 mm VAS score reduction was a slightly more conservative value. Secondary outcomes included adverse effects and overall patient satisfaction. Time to achievement of a clinically meaningful pain reduction was defined as the first time-point at which the patient reported 15 mm of pain reduction or more. Maximal pain reduction was defined as the lowest VAS score reported by the patient over the course of follow-up. The time to maximal pain reduction was defined as the time at which the patient had the lowest VAS score over the course of the 1-h follow-up.

### Statistics and data analysis

Sample size was determined based on calculations performed for previous studies examining analgesics in an ED setting [13, 14]. Random allocation was generated by the lead student researcher, and the allocation of patients was done by the student researchers at the time of patient recruitment. The trial was ended due to logistical constraints within the department after reaching the minimum sample size calculated based on the above mentioned study (which included 30+ subjects in each group).

Descriptive statistics are given as median, mean and standard deviation for continuous variables and frequency distribution for categorical variables. A comparison of treatment group means was performed using a one-way Analysis of variance (ANOVA). The multiple comparisons adjustment method of “Hochberg’s GT2” was employed for pair-wise comparisons between the groups. Dichotomous side-effects were compared using the Chi Square test or Fisher Exact test. When results of the overall test were significant, the False Discovery Rate method for adjustment of significance level was employed for pair-wise comparisons between the groups. An ANOVA with repeated measures was performed to assess the time trend and difference between the groups with respect to pain over time measurement. Statistical analysis was performed by SAS for Windows version 9.2.

## Results

### Participant flow

**Fig. 1:**
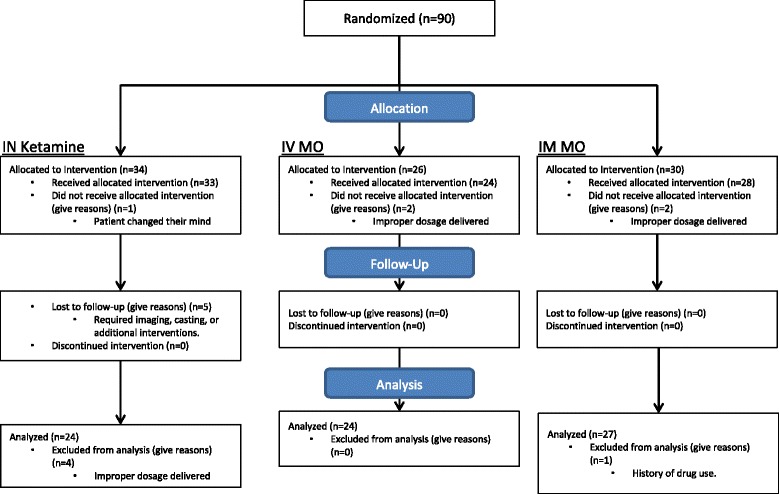
Participant Flow. The participant flow for this study shows randomization of 90 patients. Patients were lost to follow-up due to tests, imaging, and other interventions that necessitated that we halt follow-up. Patients were excluded due to dosing errors

As a convenience sample, all patients entering the orthopedic trauma bay in the ED were approached when student researchers were available. Data was collected between September 2012 and April 2014. Of those approached, 90 patients were randomized to receive IN ketamine, IV Morphine or IM morphine. Five patients were lost to follow-up in the IN ketamine group due to ED interventions such as casting or imaging, which precluded the intensive assessment required for the study. The reason for the disproportionate IN ketamine subject loss was the more rapid administration after randomization and consent. Four patients in the IN ketamine and two patients in the IM morphine group were excluded due to improper dosing. Two patients of the IV Morphine group did not receive the allocated intervention due to improper dosing, and one patient in the IN ketamine group changed their minds before the intervention. One patient in the IM MO group was excluded due to disclosure of chronic opiate use after intervention (Fig. 1).

### Baseline data

Ninety patients were recruited to the study and were randomized to the IM morphine, IN ketamine, or IV morphine study group. Fifteen patients were excluded for reasons described above, leaving 75 subjects for final analysis. Mean age of the patient population was 39.4 years (range 18–68) and 51 of the recruited patients were men (68.0 %). Table 1 shows the age and gender distribution, as well as the pre-analgesic pain level for each study group. Mean pre-analgesia VAS score for all groups was 91 mm (95 % CI 72–100 mm), with no significant difference between groups. There was no significant difference in patient age, pre-analgesic initial VAS level, or the gender distribution between the three study groups.

**Table 1:** Population statistics

	IN Ketamine (*n* = 24)	IV Morphine (*n* = 24)	IM Morphine (*n* = 27)	*p*-value
Patient Age (95 % CI)	37.9 (32.3–43.5)	42.9 (38.0–47.8)	37.7 (32.8–42.6)	0.278
Gender				
Male	17 (70.8 %)	18 (75.0 %)	16 (59.3 %)	0.455
Female	7 (29.2 %)	6 (25 %)	11 (40.7 %)	0.400
Pre-Analgesic VAS mm on a 100 mm VAS (95 % CI)	90 mm (89.7–90.3)	92 mm (91.7–92.3)	91 mm (90.7–91.3)	0.698

Shows the population statistics of the 75 patients randomized in the trial. No statistical significance was shown between the groups when analyzing patient age, gender, as well as pre-analgesic VAS levels

### Dosages

The average delivered dose of each drug was equal to the planned dose, i.e. 1.0 mg/kg for IN ketamine, 0.10 mg/kg for IV MO, and 0.15 mg/kg for IM MO.

### Comparative efficacy

Analgesic efficacy was measured via three parameters: time to onset was the primary efficacy outcome, maximal pain reduction, and the time to maximal pain reduction. Time to onset (TTO) was measured as the period of time until the patient reached a 15 mm VAS score reduction. In all patients, the average TTO was 16.7 min (95 % CI 13.7–19.7 min). Five patients overall (6.8 %) were non-responsive to treatment, meaning that they never achieved the 15 mm VAS pain reduction: 11 % of the IM morphine group, 4 % of the IN ketamine patients and 4 % of the IV morphine patients (*p* = 0.611). TTO was significantly different (*p* = 0.000) between groups (Table 2). Average TTO with IN ketamine was 14.3 min (95 % CI 9.8–18.8 min), compared to 8.9 min (95 % CI 6.6–11.2 min) for IV morphine (*p* = 0.30) and 26.0 min (95 % CI 20.3–31.7 min) for IM morphine (*p* = 0.003).

**Table 2:** Analgesic efficacy

	Time to onset Minutes (95 % CI)	Non-responders Patients (%)	Maximal pain reduction mm on a 100 mm VAS (95 % CI)	Time to max pain reduction Minutes (95 % CI)
IN Ketamine (*n* = 24)	14.3 (9.8–18.8)	1 (4 %)	56 mm VAS	40.4 min (33.9–46.9)
IV MO (*n* = 24)	8.9 (6.6–11.2)	1 (4 %)	59 mm VAS	33.4 min (26.2–40.6)
IM MO (*n* = 27)	26.0 (20.3–31.7)	3 (11 %)	48 mm VAS	46.7 min (41.1–52.3)
Aggregate	16.7 (13.7–19.7)	5 (6.8 %)	54 mm VAS	40.6 min (36.8–44.4)
*P* value IN Ketamine vs. IV MO	0.300	0.611 (DF = 2)	0.300 (DF = 2)	0.386
*P* value IN Ketamine vs. IM MO	0.003			0.441
*P* value IV MO vs. IM MO	0.000			0.019

Shows the analgesic efficacy in each group. Significant difference is shown in time to onset (time to ≥15 mm VAS reduction) between both the IN ketamine and IV MO and the IM MO group. IN Ketamine and IV MO were not different. There was no difference in the non-responder rate, nor significance in the maximal pain reduction between IN ketamine and opiate controls

Maximal pain reduction was not significantly different among the three groups, with a 56 mm, 59 mm, and 48 mm pain reduction for the IN ketamine, IV morphine, and IM morphine groups, respectively (*p* = 0.3). Time to maximal pain reduction was also not significantly different for IN ketamine versus either of the morphine groups: 40.4 min for IN ketamine (95 % CI 33.9–46.9 min), 33.4 min for IV morphine (95 % CI 26.2–40.6 min), and 46.7 min for IM morphine (95 % CI 41.1–52.3 min) (*p* = 0.019 between IM morphine and IV morphine only).

### Pharmacodynamics of pain relief

**Fig. 2:**
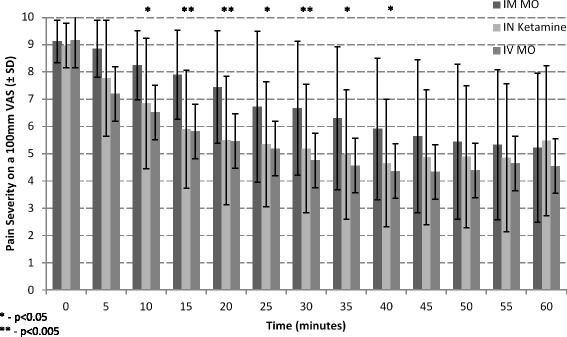
Average Pain Reduction in 5 min intervals between groups. The average VAS score for each group was graphed along with standard deviations. Intranasal Ketamine and IV morphine showed similar pain reduction, with IM morphine showing slower pain reduction over time

Pain reduction dynamics were analyzed using a 5-min pain severity sampling rate (Fig. 2; raw data presented in Additional file 1: Table S1). There was a nearly identical pain relief rate in the IV morphine and the IN ketamine groups, contrasted with the slower progression of analgesia in the IM morphine group. Significant differences in pain relief disappeared at around the 45-min mark.

### Patient satisfaction

At the end of the 1-h period of observation, patients were asked to state their satisfaction with the administered medication on a 100 mm VAS. IM morphine and IV morphine had similar results with a mean satisfaction score of 73.9 mm (95 % CI 62.9–84.9 mm), and 70.2 mm (95 % CI 55.2–85.2 mm), respectively and 58.7 mm (95 % CI 45.3–72.1 mm) for IN ketamine (*p* = 0.259).

### Adverse effects

At the end of one hour, patients were asked to report symptoms on a standardized ‘Opiate Related Symptom Distress Scale’ [11]. Of the 12 side-effects queried, only four yielded significant differences between the groups: difficulty concentrating, dizziness, confusion, and dryness of the mouth. Table 3 shows the frequency of each adverse effect in each group. There was a significant increase in reported difficulty concentrating and of dry mouth between the IN Ketamine and IV and IM morphine. There was less dizziness in patients receiving IM morphine than IN ketamine, but not IV morphine. Some patients in the IN ketamine group complained that the drug “tasted bad,” due to drug runoff into the pharynx. Finally, while no formal psychomotor testing was performed, no patient complained of hallucinations, out-of-body experiences, or emergence reactions sometimes described with IN ketamine use.

**Table 3:** Adverse Effects at 1 h

	IN Ketamine (*n* = 24)	IV MO (*n* = 24)	IM MO (*m* = 27)	IN Ketamine v. IV MO	IN Ketamine vs. IM MO
Difficulty Concentrating %	58.3 %	20.8 %	22.2 %	0.034	0.027
Dizziness %	79.2 %	50.0 %	22.2 %	0.092	<0.000
Confusion %	50.0 %	12.5 %	18.5 %	0.027	0.061
Dry-Mouth %	25.0 %	79.2 %	63.0 %	0.002	0.027

IN Ketamine showed greater frequency of difficulty in concentrating and fewer levels of dry mouth. IN ketamine and IV MO showed greater levels of dizziness. IN Ketamine showed greater levels of confusion than IV MO

### Hemodynamic and respiratory effects

As part of the safety monitoring of patients during the study, in addition to collection of patient-reported adverse events, vital signs were measured at 5 min intervals throughout the hour following administration of analgesia. Blood pressure, O_2_ saturation, respirations, and heart rate were followed. On average, patients treated with ketamine showed a larger increase and a smaller decrease in both systolic and diastolic blood pressure when compared to both morphine control groups. Respiration rate tended to increase more and decrease less than IV morphine. While these trends in vital signs are indicative of the pharmacological properties of each medication, there was no statistically significant difference in the changes of vitals for each groups (See Additional file 2: Table S2).

## Discussion

Since its formulation in 1960, ketamine has been used for a number of indications, such as sedation for painful procedures [13], endotracheal intubation and analgesia for severe pain. Due to its benign effects on the cardiovascular, respiratory and airway systems, especially important in fragile or otherwise high risk patients, it has found a growing role in the induction of anesthesia and sedation in children and in hemodynamically- compromised patients [15]. In austere conditions and in disaster and military settings, ketamine has been shown to be safe and effective for both induction and maintenance of anesthesia with or without mechanical ventilation for operations lasting up to 2 h [16]. Ketamine is widely used for sedation for painful procedures in the ED [13] as well as for analgesia in trauma [17]. In fact, low-dose ketamine has been shown effective in treating pain [18], as well as reducing dependence on opioids [19] in an ED setting. Finally, Ketamine may have neuroprotective effects in trauma as it increases cerebral blood flow [20].

Ketamine affects the limbic system and the inhibitory descending pain pathways. It has the ability to downregulate the resting state connectivity of pain-processing related structures in the brain, and upregulate the activity of pathways involved in descending inhibition of pain. It is hypothesized that during times of stress, these inhibitory pathways may be activated to reduce our perception of pain. Furthermore, ketamine’s downregulation effect within the amygdala may explain its role as a dissociative anesthetic [21].

### Efficacy of pain relief

This study attempted to assess the effectiveness of IN ketamine in the ED setting in moderate to severe pain resulting from blunt orthopedic injuries. When comparing the three measures of effectiveness, TTO, maximal pain relief, and time to maximal pain relief, IN ketamine provided equal or better pain relief compared to the standard-of-care morphine. IN ketamine produced a fast induction of pain relief, clinically comparable to an IV bolus of morphine. The ability of IN ketamine to induce significant pain relief quickly and effectively is not surprising. IN ketamine showed a 45 % bioavailability, higher than most other delivery methods [22]. IN ketamine’s clinical efficacy is likely explained by the absorption of the drug through the nasal mucosa, allowing it to act on the brain without undergoing first pass metabolism. In a study measuring efficacy and pharmacokinetics, ketamine was measured in the blood within 2 min of IN administration in far higher concentrations than the less active metabolite, norketamine [22].

Additional effectiveness parameters measured were maximal pain relief, and the time to achievement of maximal pain relief. No significant difference was seen between the three study groups on the maximal pain relief, with all three groups reducing pain very adequately by approximately 50 mm on the VAS. Pharmacokinetic studies of IN ketamine administration showed maximum concentrations in the blood at 30 min, with gradual reduction of analgesic effect starting at around 40 min [23]. In our study, IN ketamine reached the point of maximal pain reduction within an intermediate time between the IV and IM morphine. Statistically, it was not significantly different from either group. Taken together, these results indicate that IN ketamine may provide analgesia clinically equal to IV or IM morphine.

### Patient satisfaction

There was no significant difference in patient satisfaction between the groups.

### Relevance to acute trauma and military medicine

Whereas rapid and effective analgesia is of paramount importance in all medical situations, choosing the correct medication is oftentimes a challenge in severe trauma, emergency and mass casualty situations. Adverse effects such as hemodynamic instability and decreased respiratory drive require alternatives beyond opioids. IV or IM use of ketamine in the acute pre-hospital, military, or mass casualty setting has been widely discussed in the literature. A study of 40 medevac patients receiving either IM or IV ketamine for pain relief concluded that ketamine is “safe, effective, and may be more appropriate than the drugs currently used by pre-hospital providers” [24]. Whereas IV and IM ketamine have been a mainstay of analgesia care for severe pain, used by many hospital, pre-hospital and military medical providers, only recently has the IN route been actively introduced into this field. The first use of IN ketamine in the civilian pre-hospital setting, a case report from 2009, treated a 9-year-old boy with burn injuries, describing sufficient pain relief on 0.5 mg/kg of IN ketamine to dress and treat the patient’s wounds en route [25]. More recently, studies of IN ketamine in the ED have shown its efficacy and safety in a heterogeneous population of ED patients, but not specifically orthopedic and trauma [5, 6]. In a pediatric population with acute limb injuries, IN ketamine has already been shown to be equally effective and safe when compared to IN fentanyl [14]. The ease of administration and safety profile of ketamine, and the current challenges of treating pain in acute trauma patients may indicate that IN ketamine is an effective route of administration and analgesic choice for the treatment of civilians and military personnel experiencing acute trauma.

### Adverse effects

There is little evidence in the literature to suggest that IN ketamine has serious side-effects. Extensive use of IN ketamine (four sprays per hour over a period of months) resulted in anosmia in a woman suffering from breakthrough oncological pain [26]. In our study, IN ketamine showed nearly identical rates of adverse effects to that of IV and IM morphine, none of which were dangerous. Previous studies reported dizziness as the most common adverse effect with incidences as high as 52 % [5] and 31 % [6]. Nineteen patients (79 % of the ketamine group) experienced dizziness shortly after administration of IN ketamine, but it was transient in nature, universally resolving within 7–10 min, and of only mild to moderate intensity. Increased levels of difficulty concentrating and confusion in the IN ketamine population mirrored the drug’s effects as a dissociative anesthetic/analgesic. Patients reported feeling “disconnected from their pain,” or “aware but apathetic” to their pain. However, these side-effects did not preclude patients assessing their satisfaction with IN ketamine as no less than that of morphine.

### Study generalizability and limitations

We believe this study is easily generalizable to any ED, and indeed any pre-hospital or military situation. Since the pharmacokinetics of ketamine are well studied and understood, we have simply studied the safety and efficacy of the IN administration of ketamine in the acute care population. Generalizability of our data may be limited by our inclusion of only moderate-severe pain patients (80–100 mm VAS). In fact, many ED patients were excluded simply because they did not self-report pain of that intensity. Perhaps expanding to all pain levels above 60 mm VAS would be more applicable to clinical practice. Generalizability could also be increased by expanding the types of injuries studied. This study examined orthopedic limb and spine injuries; perhaps the addition of blunt chest injury and rib fractures could increase clinical utility.

Limitations of this study were mostly technical and relevant to the IN drug delivery. Atomization of IN ketamine was sometimes difficult to control using the syringe system we chose. The amount atomized with every push of the syringe was somewhat variable, leading sometimes to runoff into the pharynx. Studies of IN delivery show that volumes greater than 300 μl per nostril may be difficult to absorb through the nasal mucosa, leading to reduced drug absorption [1]. Furthermore, IN dosing has been shown to be variable, indicating that future work should focus on identifying the proper doses and drug concentrations as well as delivery devices, for optimal absorption and efficacy. Our follow-up duration of 1 h was limited, though geared towards the primary study endpoints. An additional limitation of this study was the lack of researcher blinding. While any study of this nature would benefit from blinding, the different routes of administration made blinding complicated in a busy ED setting without the use of sham IV/IM/IN drug administration. A blinded study is already underway in our department, looking at longer - term effects of IN ketamine as well as treatment for a more diverse patient population. Finally, while we believe that there is good generalizability and external validity to this study, due to the clinically relevant setting we used, larger studies, looking at more diverse causes of pain are still needed.

## Conclusions

IN ketamine showed efficacy and safety comparable to the current standard-of-care for acute traumatic pain in the ED, IM and IV morphine. IN ketamine provided rapid pain relief without causing hemodynamic instability or respiratory side-effects, was easy to administer and may thus be an important option for ED settings, pre-hospital trauma management, as well as in battlefield and disaster medicine. Further studies are needed in order to define optimal concentrations and doses of IN ketamine, as well as improved delivery devices able to deliver a controlled quantity of ketamine to the patient.

## Additional files

Additional file 1: Table S1. Raw data table for Fig. 2. This table includes the mean and SD of VAS for each group at each time point. (DOCX 21 kb)
Additional file 2: Table S2. Hemodynamic and Respiratory Data. This Table includes an average of the maximal and minimal changes in vital signs for patients in each treatment group. (DOCX 14 kb)

## Abbreviations

ANOVA: Analysis of variance
ASA: American Society of Anesthesiologists
ED: Emergency Department
GCS: Glasgow Coma Score
IDF: Israeli Defense Force
TAMC: Tel Aviv Medical Center
TTO: Time to onset
VAS: Visual analog scale

## References

[1] Wolfe TR, Braude DA (2010). Intranasal medication delivery for children: a brief review and update. Pediatrics.

[2] Karlsen AP, Pedersen DM, Trautner S, Dahl JB, Hansen MS (2014). Safety of Intranasal Fentanyl in the Out-of-Hospital Setting: A Prospective Observational Study. Ann Emerg Med.

[3] Veldhorst-Janssen NM, Fiddelers AA, van der Kuy PH, Neef C, Marcus MA (2009). A review of the clinical pharmacokinetics of opioids, benzodiazepines, and antimigraine drugs delivered intranasally. Clin Ther.

[4] Schwartz RB, Charity BM (2001). Use of night vision goggles and low-level light source in obtaining intravenous access in tactical conditions of darkness. Mil Med.

[5] Andolfatto G, Willman E, Joo D, Miller P, Wong W-B, Koehn M (2013). Intranasal ketamine for analgesia in the emergency department: a prospective observational series. Acad Emerg Med.

[6] Yeaman F, Meek R, Egerton-Warburton D, Rosengarten P, Graudins A (2014). Sub-dissociative-dose intranasal ketamine for moderate to severe pain in adult emergency department patients. Emerg Med Australas.

[7] Bourgoin A, Albanese J, Wereszczynski N, Charbit M, Vialet R, Martin C (2003). Safety of sedation with ketamine in severe head injury patients: comparison with sulfentanyl. Crit Care Med.

[8] Cohen L, Athaide V, Wickham ME, Doyle-Waters MM, Rose NGW, Hohl CM. The Effect of Ketamine on Intracranial and Cerebral Perfusion Pressure and Health Outcomes: A Systematic Review. Annals of Emergency Medicine 2014; published online July 22, 2014 ahead of print. 10.1016/j.annemergmed.2014.06.018 25064742

[9] Kolenda H, Gremmelt A, Rading S, Braun U, Markakis E (1996). Ketamine for analgosedative therapy in intensive care treatment of head-injured patients. Acta Neurochir (Wien).

[10] Mayberg TS, Lam AM, Matta BF, Domino KB, Winn HR (1995). Ketamine does not increase cerebral blood flow velocity or intracranial pressure during isoflurane/nitrous oxide anesthesia in patients undergoing craniotomy. Anesth Analg.

[11] Apfelbaum JL, Gan TJ, Zhao S, Hanna DB, Chen C (2004). Reliability and validity of the perioperative opioid-related symptom distress scale. Anesth Analg.

[12] Powell CV, Kelly AM, Williams A (2001). Determining the minimum clinically significant difference in visual analog pain score for children. Ann Emerg Med.

[13] Uri O, Behrbalk E, Haim A, Kaufman E, Halpern P (2011). Procedural sedation with propofol for painful orthopaedic manipulation in the emergency department expedites patient management compared with a midazolam/ketamine regimen: a randomized prospective study. J Bone Joint Surg Am.

[14] Graudins A, Meek R, Egerton-Warburton D, Oakley E, Seith R (2015). The PICHFORK (Pain in Children Fentanyl or Ketamine) trial: a randomized controlled trial comparing intranasal ketamine and fentanyl for the relief of moderate to severe pain in children with limb injuries. Ann Emerg Med.

[15] Cravero JP, Havidich JE (2011). Pediatric sedation--evolution and revolution. Paediatr Anaesth.

[16] Bonanno FG (2002). Ketamine in war/tropical surgery (a final tribute to the racemic mixture). Injury.

[17] Jennings PA, Cameron P, Bernard S (2011). Ketamine as an analgesic in the pre-hospital setting: a systematic review. Acta Anaesthesiol Scand.

[18] Richards JR, Rockford RE (2013). Low-dose ketamine analgesia: patient and physician experience in the ED. Am J Emerg Med.

[19] Beaudoin FL, Lin C, Guan W, Merchant RC (2014). Low-dose ketamine improves pain relief in patients receiving intravenous opioids for acute pain in the emergency department: results of a randomized, double-blind, clinical trial. Acad Emerg Med.

[20] Porter K (2004). Ketamine in prehospital care. Emerg Med J.

[21] Niesters M, Khalili-Mahani N, Martini C, Aarts L, van Gerven J, van Buchem MA (2012). Effect of subanesthetic ketamine on intrinsic functional: a placebo-controlled functional magnetic resonance imaging study in healthy male volunteers. Anesthesiology.

[22] Yanagihara Y, Ohtani M, Kariya S, Uchino K, Hiraishi T, Ashizawa N (2003). Plasma concentration profiles of ketamine and norketamine after administration of various ketamine preparations to healthy Japanese volunteers. Biopharm Drug Dispos.

[23] Carr DB, Goudas LC, Denman WT, Brookoff D, Staats PS, Brennen L (2004). Safety and efficacy of intranasal ketamine for the treatment of breakthrough pain in patients with chronic pain: a randomized, double-blind, placebo-controlled, crossover study. Pain.

[24] Svenson JE, Abernathy MK (2007). Ketamine for prehospital use: new look at an old drug. Am J Emerg Med.

[25] Reid C, Hatton R, Middleton P (2011). Case report: prehospital use of intranasal ketamine for paediatric burn injury. Emerg Med J.

[26] Mayell A, Natusch D (2009). Anosmia - a potential complication of intranasal ketamine. Anaesthesia.

