# Supplementary material for: Intranasal ketamine for acute traumatic pain in the Emergency Department: a prospective, randomized clinical trial of efficacy and safety
Source: BMC Emerg Med. 2016 Nov 9;16:43. doi: 10.1186/s12873-016-0107-0 (PMC5103427; doi:10.1186/s12873-016-0107-0)
Supplement: Additional file 1: Table S1. — Raw data table for Fig. 2. This table includes the mean and SD of VAS for each group at each time point. (DOCX 21 kb) [file 12873_2016_107_MOESM1_ESM.docx]

Additional file 1: Table S1: Raw data table for Figure 2.

|  | 0 min | 5 min | 10 min | 15 min | 20 min | 25 min | 30 min | 35 min | 40 min | 45 min | 50 min | 55 min | 60 min |
| --- | --- | --- | --- | --- | --- | --- | --- | --- | --- | --- | --- | --- | --- |
| IN Ket VAS | 9.0 | 7.8 | 6.8 | 5.9 | 5.5 | 5.3 | 5.2 | 5.0 | 4.7 | 4.9 | 4.9 | 4.9 | 5.5 |
| IN Ket SD | 0.93 | 1.92 | 2.14 | 2.48 | 2.50 | 2.60 | 2.60 | 2.50 | 2.31 | 2.49 | 2.46 | 2.52 | 2.66 |
| IV MO VAS | 9.2 | 7.2 | 6.5 | 5.8 | 5.5 | 5.2 | 4.8 | 4.6 | 4.4 | 4.3 | 4.4 | 4.7 | 4.6 |
| IV MO SD | 0.82 | 2.13 | 2.39 | 2.16 | 2.36 | 2.29 | 2.35 | 2.38 | 2.34 | 2.48 | 2.60 | 2.72 | 2.75 |
| IM MO VAS | 9.1 | 8.9 | 8.3 | 7.9 | 7.4 | 6.7 | 6.7 | 6.3 | 5.9 | 5.6 | 5.4 | 5.3 | 5.2 |
| IM MO SD | 0.78 | 1.04 | 1.26 | 1.63 | 2.06 | 2.77 | 2.46 | 2.62 | 2.60 | 2.80 | 2.85 | 2.76 | 2.74 |
| p-value | 0.9482 | 0.0246* | 0.0111* | 0.0006** | 0.0011** | 0.0224* | 0.0048** | 0.0131* | 0.0282* | 0.1022 | 0.2365 | 0.5223 | 0.3194 |
