# Supplementary material for: Intranasal ketamine for acute traumatic pain in the Emergency Department: a prospective, randomized clinical trial of efficacy and safety
Source: BMC Emerg Med. 2016 Nov 9;16:43. doi: 10.1186/s12873-016-0107-0 (PMC5103427; doi:10.1186/s12873-016-0107-0)
Supplement: Additional file 2: Table S2. — Hemodynamic and Respiratory Data. This Table includes an average of the maximal and minimal changes in vital signs for patients in each treatment group. (DOCX 14 kb) [file 12873_2016_107_MOESM2_ESM.docx]

Additional file 2: Table S2: Hemodynamic and Respiratory Data

|  | IN Ketamine | IM MO | IV MO |
| --- | --- | --- | --- |
| HR Increase (bpm) (95%CI) | 8.4 (5.5-11.3) | 7.2 (4.5-9.8) | 6.3 (3.2-9.5) |
| HR Decrease (bpm) (95%CI) | 6.4 (4.8-8.0) | 5.2 (2.8-7.6) | 7.5 (4.7-10.2) |
| Systolic BP Increase (mmHg) (95%CI) | 23.5 (17.0-30.0) | 14.1 (9.6-18.6) | 12.8 (8.2-17.4) |
| Systolic BP Decrease (mmHg) (95%CI) | 6.8 (1.4-12.2) | 10.0 (4.7-15.2) | 13.5 (9.4-17.5) |
| Diastolic BP Increase (mmHg) (95%CI) | 17.4 (13.0-21.8) | 11.9 (7.9-15.8) | 10.2 (6.4-13.9) |
| Diastolic BP Decrease (mmHg) (95%CI) | 5.6 (1.5-9.7) | 11.4 (6.3-16.6) | 9.7 (5.6-13.8) |
| Respiratory Rate Increase (Resp/min) (95%CI) | 2.6 (1.7-3.4) | 1.6 (0.9-2.2) | 1.1 (0.03-2.2) |
| Respiratory Rate Decrease (Resp/min) (95%CI) | 3.5 (2.4-4.6) | 3.5 (2.3-4.7) | 4.5 (3.4-5.6) |
| O2 Sat Increase (%sat)  (95%CI) | 0.01 (0.01-0.014) | 0.01 (0.01-0.02) | 0.01 (0.01-0.02) |
| O2 Sat Decrease (%sat)  (95%CI) | 0.02 (0.02-0.03) | 0.05 (0.0-0.1) | 0.02 (0.01-0.02) |

The table shows an average of the maximal and minimal changes in vital signs for patients in each treatment group. IN Ketamine showed greater increases and lesser decreases in both systolic and diastolic blood pressure when compared to IV or IM morphine. Furthermore, IN ketamine showed greater respiratory rate increases than both control groups and lesser respiratory depression than IV morphine. While these trends seem in line with the expected pharmacological effects of each analgesic, they did not reach statistical significance.
